# A small number of residual teeth after the mandibular resection of oral cancer is associated with titanium reconstruction plate exposure

**DOI:** 10.1002/cre2.208

**Published:** 2019-06-07

**Authors:** Hiromi Hirohata, Toru Yanagawa, Shohei Takaoka, Kenji Yamagata, Kaoru Sasaki, Yoichiro Shibuya, Fumihiko Uchida, Satoshi Fukuzawa, Katsuhiko Tabuchi, Shogo Hasegawa, Naomi Ishibashi‐Kanno, Mitsuru Sekido, Hiroki Bukawa

**Affiliations:** ^1^ Oral and Maxillofacial Surgery, Clinical Sciences, Graduate School of Comprehensive Human Sciences University of Tsukuba Tsukuba Japan; ^2^ Department of Oral and Maxillofacial Surgery Tsukuba Central Hospital Ushiku Japan; ^3^ Department of Oral and Maxillofacial Surgery, Faculty of Medicine University of Tsukuba Tsukuba Japan; ^4^ Department of Oral and Maxillofacial Surgery Ibaraki Prefectural Central Hospital Kasama Japan; ^5^ Department of Plastic and Reconstruction Surgery, Faculty of Medicine University of Tsukuba Tsukuba Japan; ^6^ Department of Neurohealth Innovation, Institute for Biomedical Sciences Interdisciplinary Cluster for Cutting Edge Research, Department of Molecular & Cellular Physiology Shinshu University School of Medicine Matsumoto Japan; ^7^ Department of Oral and Maxillofacial Surgery, Department of Maxillofacial Surgery, School of Dentistry Aichi Gakuin University Nagoya Japan

**Keywords:** mandibular reconstruction, neoplasm, reconstruction plate

## Abstract

**Objective:**

Reconstruction plates are used to treat patients with a segmental mandibular defect after oral cancer surgery. Reconstruction plate failure analysis has rarely focused on occlusion, which conducts a mechanical force to the mandible and the plate. To determine the prognostic factors, we retrospectively evaluated patients who underwent reconstruction of a mandibular segmental defect with a reconstruction plate and assessed the number of residual paired teeth.

**Material and Methods:**

From among 390 patients with oral cancer who visited University of Tsukuba Hospital (Tsukuba, Japan) between 2007 and 2017, we selected and analyzed the data of 37 patients who underwent segmental resection of the mandible and reconstruction with reconstruction plates. Prognostic factors evaluated were patient age, sex, TNM classification, plate manufacturer, treatment with radiotherapy or chemotherapy, whether the patient had diabetes or smoked, and whether the patient had a small number of residual paired teeth, plate length, and use of a fibular‐free flap. Among these 37 patients, eight reconstruction plates had intraoral or extraoral exposure and were removed in 5 years.

**Results:**

Kaplan–Meier and log‐rank analyses revealed that the prognosis for the 5‐year plate exposure‐free rate was significantly poorer for patients with a small number of residual teeth than for patients with no teeth or those with a large number of residual teeth (.01). Univariate Cox regression analysis revealed that a small number of residual teeth was a significant prognostic factor in the loss of a reconstruction plate (hazard ratio: 5.63; 95% confidence interval [1.10, 25.85]; .04).

**Conclusions:**

A small number of residual teeth after the segmental resection of oral cancer is significantly involved in reconstruction plate survival and may be important in predicting reconstruction plate prognosis.

## 1 BACKGROUND

Treating advanced oromandibular cancer often requires resecting mandibular and alveolar bones and soft tissues such as the oral mucosa, muscles, or external skin of the mandible. Mandibular reconstruction with a plate, with or without a vascularized free flap, is a common treatment to deliver acceptable function and cosmesis to maintain the postoperative quality of life. The role of the reconstruction plate is very important because an unreconstructed mandibular defect may cause deformation of the remaining mandible in association with several problems for the patients such as insufficient sealing of the lips and feeding (van der Rijt, Noorlag, Koole, Abbink, & Rosenberg, 2015). However, many postsurgery complications occur with reconstruction plates, and the rates of reported complications with reconstruction plates range from 28% to 39% and include loosening of the osteosynthesis screws, plate fracture, and intraoral or extraoral exposure or infection (Kammerer, Klein, Moergel, Gemmel, & Draenert, 2014; Maurer, Eckert, Kriwalsky, & Schubert, 2010). A recent systematic review and meta‐analysis (Sadr‐Eshkevari et al., 2013) demonstrated that the failure rate was 30.8% at the 32‐month follow‐up and discussed the risk factors of reconstruction plate failure from various viewpoints. Many reports discuss the influencing factors for reconstruction plate survival and list many factors such as physical factors such as whether an individual has diabetes mellitus (van der Rijt et al., 2015), smoking habit (Maurer et al., 2010; van der Rijt et al., 2015), whether an individual has had a blood transfusion (Fanzio et al., 2015), and surgical infection site (Wood, Shinn, Amin, Rohde, & Sinard, 2018), hardware factors such as plate length and location of a mandibular defect (Arden, Rachel, Marks, & Dang, 1999; Ettl et al., 2010; Mariani, Kowalski, & Magrin, 2006; Okura, Isomura, Iida, & Kogo, 2005; Poli, Ferrari, Bianchi, & Sesenna, 2003; Prasad et al., 2018; Shibahara, Noma, Furuya, & Takaki, 2002), and treatment factors such as radiation therapy and chemotherapy (Okura et al., 2005; Ryu et al., 1995; Shibahara et al., 2002; Wang, Zhang, & Mendenhall, 2005). Most reports have focused on the shape of the mandibular bone defect after resection and hardware‐related complications; however, few reports have analyzed risk factors for plate failure from the viewpoint of dental occlusion (i.e., the mechanical force exerted on the reconstructed mandible and plate).

The maxillomandibular occlusal state differs depending on the relationship between the upper and lower teeth. Disharmony of chewing motion after surgery is caused by the resection of the mandibular bone and the masticatory and facial muscles. The load of the stress then alters depending on whether a stable stop position of occlusion does or does not exist. In this study, we investigated the predictive factors of reconstruction plate exposure by using the number of paired teeth as a variable to define prognostic factors for reconstruction plate loss and to improve future treatment planning.

## 2 METHODS

### 2.1 Patient characteristics

From among 390 patients with oral cancer who consulted serially in the Department of Oral and Maxillofacial Surgery at the University of Tsukuba Hospital (Tsukuba City, Japan) between 2007 and 2017, out of 390 oral cancers, 134 cases include mandibular regions (lower gingiva, buccal mucosa, and so on) and 77 patients received surgery. Among them, we selected 37 patients who underwent segmental resection of mandibular and reconstruction with reconstruction plates and analyzed the data. The patients included 21 men and 16 women with a mean age (standard deviation) of 65.8 (10.4) years. Each case of oral cancer was staged, using the International Union against Cancer system (Sobin, Wittekind, & Gospodorowicz, 2009). Prognostic factors determined from the patients' medical records were as follows: the patients' age and sex; stage of the cancer; number of screws; number of residual paired teeth; plate manufacturer (MODUS 2.5‐mm locking reconstruction plate [Mediartis, Basel, Switzerland]; Lorenz 2.4‐mm locking reconstruction plate [Biomet, Jacksonville, USA]; CMF 2.4‐mm titanium locking reconstruction plate [Synthes GmbH, Oberdorf Switzerland]); type of flap (i.e., no flap, fibular‐free flap, rectus abdominis myocutaneous flap, nasolabial flap, or latissimus dorsi flap); infection site; stage; whether the patient had diabetes or smoked; and whether the patient had received chemotherapy or radiotherapy. Residual paired teeth meant teeth that can stably maintain a vertical dimension of occlusion. Therefore, if the mobility of the opposite tooth was substantial, which included mobility occurring with severe periodontitis, we did not include it as a residual paired tooth. By using Eichner's index, which is a system for classification of partial edentulous arches based on occlusal contact between in the premolar and molar regions, ll dental arches that had residual paired teeth were classified as Eichner's index B3 (Eichner, 1995), and dental arches that had no residual paired teeth were classified as Eichner's index C1–C3 (Eichner, 1995). The clinical features of the patients are presented in Table 1. The patients were treated by surgical excision alone (23 patients) or by surgical excision plus chemoradiotherapy (14 patients). The 14 patients were treated by linear accelerator radiosurgery (mean dose, 52.4 ± 9.4 Gy; range, 37.3–60 Gy). The study protocol was reviewed and approved by the Research Ethics Committee of the University of Tsukuba (Tsukuba, Japan; approval no., H29‐258).

**Table 1:** Clinical feature of patients

Variables		
Age	Mean = 65.83	*SD* = 10.42
Sex		
Male	21	
Female	16	
Plate length (mm)	Median = 128	Range = 72–240
Number of screws	Median = 8	Range = 4–14
Number of residual paired teeth	Median = 5	Range = 0–12
Manufacturer		
Mediartis MODUS 2.5‐mm locking reconstruction plate	16	
Biomet Lorenz 2.4‐mm locking reconstruction plate	18	
Synthes CMF 2.4‐mm Titanium locking reconstruction plate	3	
Flap		
No flap	12	
Fibular‐free flap	19	
Rectus abdominis myocutaneous flap	4	
Nasolabial flap	1	
Latissimus dorsi flap	1	
Site infection		
Yes	5	
No	32	
TNM classification stage		
1	3	
2	3	
3	4	
4a	26	
4b	1	
Diabetes mellitus		
Yes	8	
No	29	
Smoking		
Yes	18	
No	19	
Chemotherapy		
Yes	12	
No	25	
Radiation therapy		
Yes	14	
No	23	

### 2.2 STATISTICAL ANALYSIS

For the univariate analysis, we used Kaplan–Meier analysis, evaluated by the log‐rank test, and the Cox proportional hazard regression model. Statistical analyses were conducted using the software package JMP 12.0.1 for Mac (SAS Institute Inc., Cary, NC, USA).

## 3 RESULTS

### 3.1 Univariate analysis of the 5‐year exposure‐free rate based on Kaplan–Meier analysis and the log‐rank test

**Figure 1:**
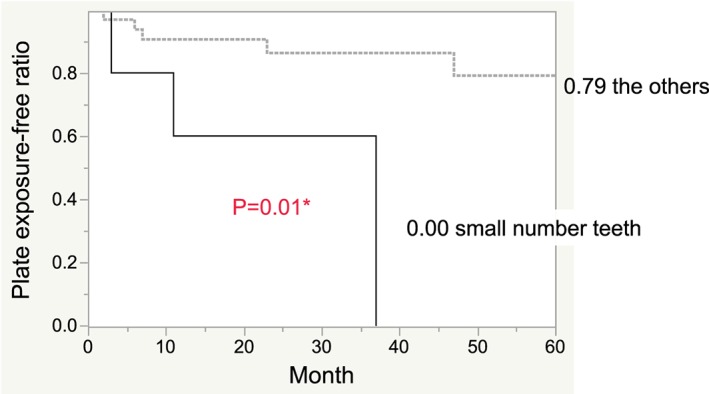
Kaplan–Meier estimates of the plate exposure‐free rate based on the number of teeth. The solid line indicates the small number of teeth group; the dotted line indicates the other group (i.e., null teeth group + large number of teeth group; P=0.01, based on the log‐rank test)

Kaplan–Meier analysis was used to estimate each factor's 5‐year exposure‐free rate (i.e., 5‐year survival rate of the reconstruction plate). During this term, reconstruction plate exposure occurred in eight (21.6%) of 37 patients. The overall 5‐year exposure‐free rate was 0.71, as estimated using Kaplan–Meier analysis. Table 2 presents the 5‐year exposure‐free rate and log‐rank test results based on Kaplan–Meier analysis. Figures 1 and S1–S13 present the survival curves for each factor. The 5‐year exposure‐free rate was significantly different between the null tooth group (i.e., zero teeth), small number of teeth group (i.e., 1–5 residual paired teeth), and large number of teeth group (i.e., six residual paired teeth or more); the rate was 0.81, 0.00, and 0.79 for the null group, small number of teeth group, and large number of teeth group, respectively (*p* = .04; Figure S5). We then divided the tooth groups into two groups: (Arden et al., 1999) small number of teeth group and (Coletti, Ord, & Liu, 2009) large number of teeth group and null teeth group combined (i.e., other group). We divided the type of flap into two groups: (Arden et al., 1999) the fibular‐free flap group and (Coletti et al., 2009) other type of flap group. The TNM classification stage was classified into two groups: (Arden et al., 1999) Stages 1–3 group and (Coletti et al., 2009) Stages 4a and 4b group. The 5‐year exposure‐free rate was insignificantly associated with age (≥65 years, 0.71; <65 years, 0.66; *p* = .29), sex (male, 0.65; female, 0.80; *p* = .24), plate length (<128 mm, 0.74; ≥128 mm, 0.66; *p* = .83), number of screws (≥8, 0.74; <8, 0.66; *p* = .19), manufacturer (Mediartis, 0.64; Biomet + Synthes, 0.85; *p* = .44), type of flap (fibular‐free flap, 0.74; other flap type, 0.67; *p* = .67), infection site (yes, 0.80; no, 0.68; *p* = .84), cancer stage (1–3, 0.76; 4a and 4b, 0.69; *p* = .59), diabetes mellitus (yes, 0.73; no, 0.72; *p* = .71), smoking status (yes, 0.80; no, 0.65; *p* = .65), chemotherapy (yes, 0.49; no, 0.81; *p* = .24), and radiation therapy (yes, 0.58; no, 0.79; *p* = .46). However, a significant difference in the 5‐year exposure‐free rate existed between the small number of teeth group and the other group (small number of teeth group, 0.00; other group, 0.79; *p* = .01).

**Table 2:** 5‐year exposure free rate by Kaplan–Meier analysis and log‐rank test

Variable	5‐year plate exposure free ratio	Log‐rank test (p value)
Age		
≦65	0.71	.29
<65	0.66	
Sex		
Male	0.65	.24
Female	0.80	
Plate length		
≦128	0.74	.83
<128	0.66	
Number of screws		
≦8	0.74	.19
<8	0.66	
Number of residual paired teeth		
Small tooth number group (<0, ≦5)	0.00	.01
The others	0.79	
Manufacturer		
Mediartis	0.64	.44
Biomet + Synthes	0.85	
Flap		
Fibular‐free flap	0.74	.67
The others	0.67	
Site infection		
Yes	0.80	.84
No	0.68	
TNM classification Stage		
1–3	0.76	.59
4a,b	0.69	
Diabetes mellitus		
Yes	0.73	.71
No	0.72	
Smoking		
Yes	0.80	.65
No	0.65	
Chemotherapy		
Yes	0.49	.24
No	0.81	
Radiation therapy		
Yes	0.58	.46
No	0.79	

### 3.2 Univariate analysis by using the Cox proportional hazard regression model

Table 3 presents the univariate analysis results based on the Cox proportional hazard regression model. The univariate analysis of each factor revealed a significant difference between the small number of teeth group and the other group (hazard ratio, 5.63; 95% confidence interval [1.10, 25.85]; *p* = .04). There was no significant association with age, sex, plate length, number of screws, manufacturer, type of flap, infection site, stage, diabetes mellitus, smoking, chemotherapy, and radiation therapy.

**Table 3:** Univariate analysis of plate exposure by using Cox's regression analysis

Variable	Hazard ratio	95% confidence interval	p value
Age			
65≦	0.43	[0.06, 1.88]	.27
<65			
Sex			
Male	2.55	[0.58, 17.52]	.22
Female			
Plate length			
≦128	0.85	[0.19, 3.76]	.83
128<			
Number of screws			
≦8	0.73	[0.66, 0.17]	.66
<8			
Number of residual paired teeth			
<0, ≦5	5.63	[1.10, 25.85]	.04
The others			
Manufacturer			
Mediartis	1.77	[0.41, 8.85]	.44
Biomet + Synthes			
Flap			
Fibular‐free flap	0.76	[0.18, 3.23]	.70
The others			
Site infection			
Yes	0.80	[0.04, 4.55]	.83
No			
TNM classification stage			
1–3	0.64	[0.09, 2.84]	.58
4a,b			
Diabetes mellitus			
Yes	1.36	[0.20, 6.06]	.72
No			
Smoking			
Yes	1.14	[0.27, 4.83]	.86
No			
Chemotherapy			
Yes	2.27	[0.53, 9.71]	.26
No			
Radiation therapy			
Yes	1.68	[0.39, 7.17]	.47
No			

## 4 DISCUSSION

In the present study, we retrospectively investigated the prognostic factors for the loss of reconstruction plates in patients with a mandibular segmental defect. In univariate analysis, conducted using Kaplan–Meier analysis evaluated by log‐rank test, the 5‐year exposure‐free rate showed no significant association with the clinical factors (i.e., age, sex, plate length, number of screws, manufacturer, type of flap, infection site, diabetes mellitus, smoking, chemotherapy, and radiation therapy). However, the 5‐year exposure‐free rate of the small number of teeth group was significantly less than that of the other group (small number of teeth group, 0.00; the other group, 0.79; *p* = .011). The univariate analysis using the Cox proportional hazard regression model revealed a significant association in the small number of teeth group (hazard ratio, 5.63; 95% confidence interval [1.10, 25.85]; *p* = .04). These findings indicated that a small number of teeth may be a poor predictive factor for reconstruction plate survival.

A recent systemic review and meta‐analysis of alloplastic mandibular reconstruction has been reported (Sadr‐Eshkevari et al., 2013). In this study, the failure rate of the reconstruction plate was 30.8% (interquartile range, 11.7–48.1%). In our study, reconstruction plate exposure occurred in eight (21.6%) of 37 patients, and the overall 5‐year exposure‐free rate was 70.6%. Thus, our clinical outcome was comparable with previous reports (Arden et al., 1999; Fanzio et al., 2015; Maurer et al., 2010; Okura et al., 2005). In various studies, many predictive factors have been presented, and physical factors (Fanzio et al., 2015; Maurer et al., 2010; van der Rijt et al., 2015; Wood et al., 2018), hardware factors (Arden et al., 1999; Ettl et al., 2010; Mariani et al., 2006; Okura et al., 2005; Poli et al., 2003; Prasad et al., 2018; Shibahara et al., 2002), and treatment factors (Okura et al., 2005; Ryu et al., 1995; Shibahara et al., 2002; Wang et al., 2005) have been reported. Among these factors, plate length and the location of a mandibular defect influenced the prognosis of plate survival. Many reports show various predictive factors of the reconstruction plate; however, only a few reports are presented from the viewpoint of occlusion. Kammerer et al. (2014) presented the concept of the tooth unit. A small tooth unit is associated with a poor survival rate of reconstruction plates. Coletti et al. (2009) demonstrated that postoperative dentition is associated with poor prognosis of the plates. These studies showed the number of teeth was an indicator of length of survival of the plate, but the studies did not discuss occlusion, which determines the mechanical stress applied to the resected mandible and reconstruction plate.

Occlusion involves bringing the opposing surfaces of the teeth of the two jaws into contact in association with complicated chewing motion. During surgery for oromandibular cancer, surgeons resect the mandibular bone and peripheral soft tissues, including the masticatory and facial muscles. Furthermore, resection includes sensory nerves such as a branch of the trigeminal nerve. A disharmony in chewing motion may produce an unexpected overload on the reconstruction plate owing to irregular movement and a patient's unawareness of the stress overload. The maxillomandibular occlusal state differs based on the relationship between the upper and lower teeth. The stress load is altered if the area of the stable stop position that bears the occlusal force is different. Markwardt, Pfeifer, Eckelt, and Reitemeier (2007) attempted to analyze the risk factors for complications by using Eichner's classification; however, they could not find a significant difference among the factors examined. Markwardt did not conduct a long‐term observation; therefore, they may not have been able to determine a significant difference. However, some reports (Hoefert & Taier, 2018; Park, Lee, & Noh, 2018; Yi et al., 1999) present analysis results from a biomechanical viewpoint and demonstrate that reconstruction plates and screws are often subjected to excessive stress produced by functional loading, moment, and shear forces. From these reports, the reconstruction plate bridging mandibular bone can be estimated to burden excessive load by the shape of bone defect and the loading points (Hoefert & Taier, 2018; Park et al., 2018; Yi et al., 1999). In particular, Park et al., 2018 divided patients into three groups—unilateral molar clenching, group function clenching, and incisal clenching—and measured the von Mises stress, a value used to determine if a given material will yield or fracture, occurring on the reconstruction plate. The maximum von Mises stress on the reconstruction plate of patients with unilateral molar clenching was larger than in that of group function clenching (Park et al., 2018). Unilateral molar clenching group in their analysis resembles the small number of teeth group in our study, and the group function clenching in their study resembles large number of teeth group in our study.

Our results may theoretically be supported from the viewpoint of biomechanical analysis. However, there is no point in loading an occlusal force in an edentulous jaw. The mechanical stress caused by occlusal movement such as twisting torsions may be reduced.

Our findings showed a close relationship between a small number of residual teeth and reconstruction plate prognosis. To clarify this causal relationship, a biomechanical analysis of the stress load to the mandible and reconstruction plate is needed. However, if this problem can be clarified, the prognosis of the reconstruction plate can be determined, and countermeasures to reduce the stress can be established. The stress generated by occlusion should be analyzed from multiple perspectives in future studies.

## 5 CONCLUSIONS

The 5‐year exposure‐free survival of a mandibular plate was poorer for patients with a small number of residual paired teeth than for patients with no teeth or those with a large number of residual paired teeth. Thus, stress burden on the reconstruction plate appears to differ based on the number of residual paired teeth. To accurately specify the cause of this stress burden and utilize it for treatment, future in vivo studies on the stress load on the reconstruction plate are needed.

## CONFLICT OF INTEREST

The authors declare that they have no competing interests.

## Supporting information

Data S1. Supporting information
