# Supplementary material for: A small number of residual teeth after the mandibular resection of oral cancer is associated with titanium reconstruction plate exposure
Source: Clin Exp Dent Res. 2019 Jun 7;5(5):469–75. doi: 10.1002/cre2.208 (PMC6820575; doi:10.1002/cre2.208)
Supplement: Supplementary file 1 — Data S1. Supporting information [file CRE2-5-469-s001.pdf]

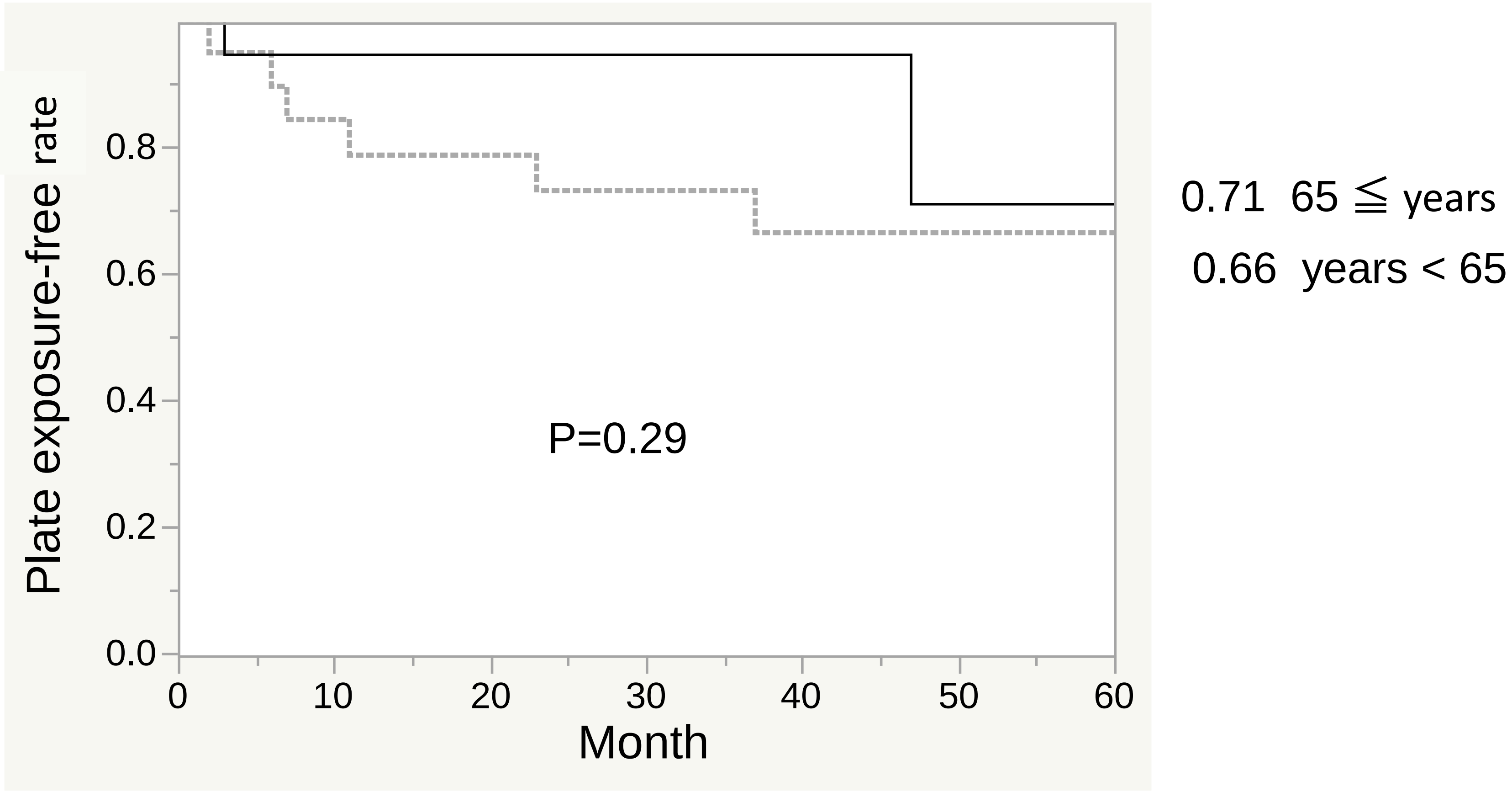
Plate exposure-free rate
0.8
0.6
0.4
0.2
0.0
0
10
20
30
40
50
60
Month
P=0.29
0.71 65 ≦ years
0.66 years < 65

Figure S1

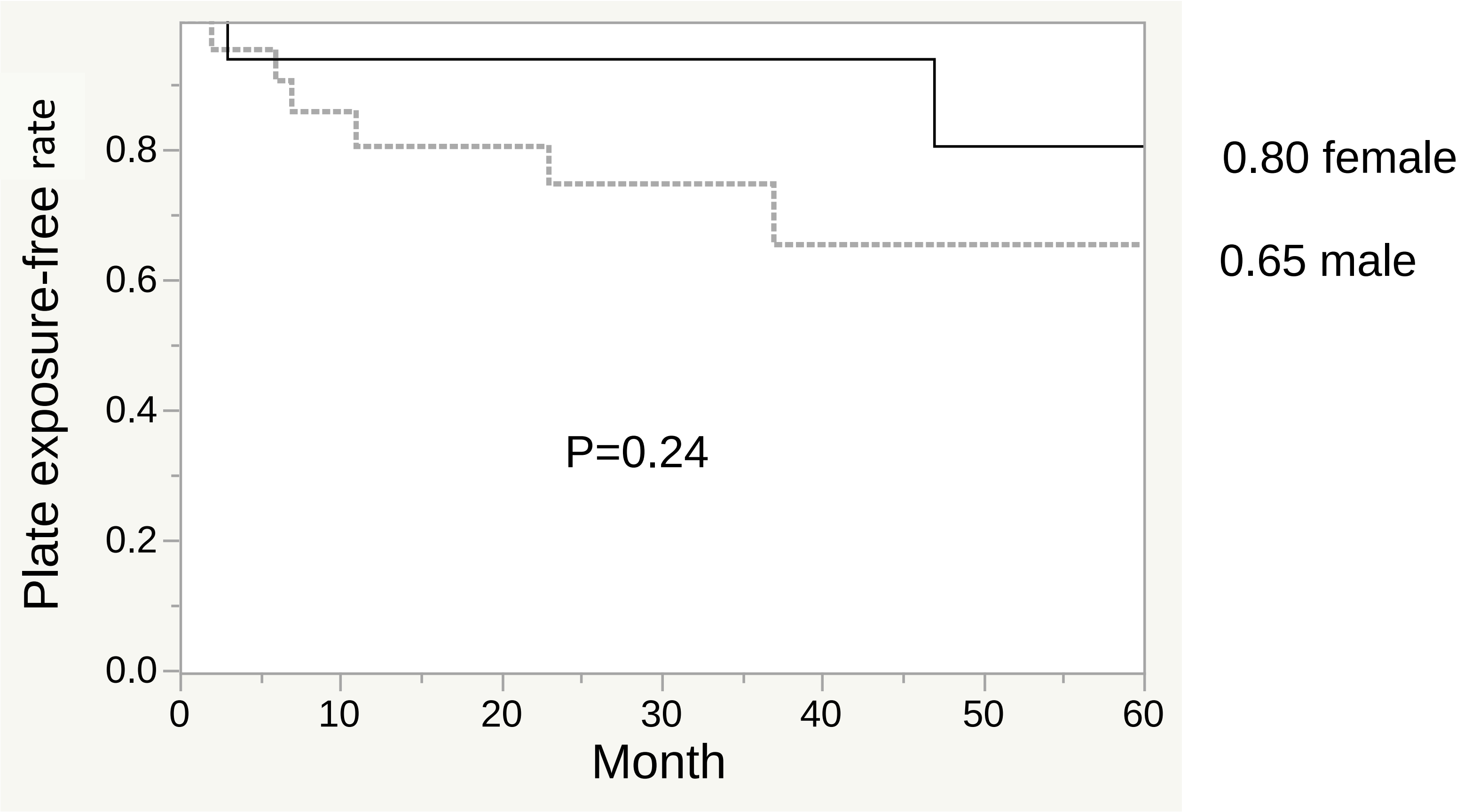
Plate exposure-free rate
0.8
0.6
0.4
0.2
0.0
0
10
20
30
40
50
60
Month
P=0.24
0.80 female
0.65 male

Figure S2

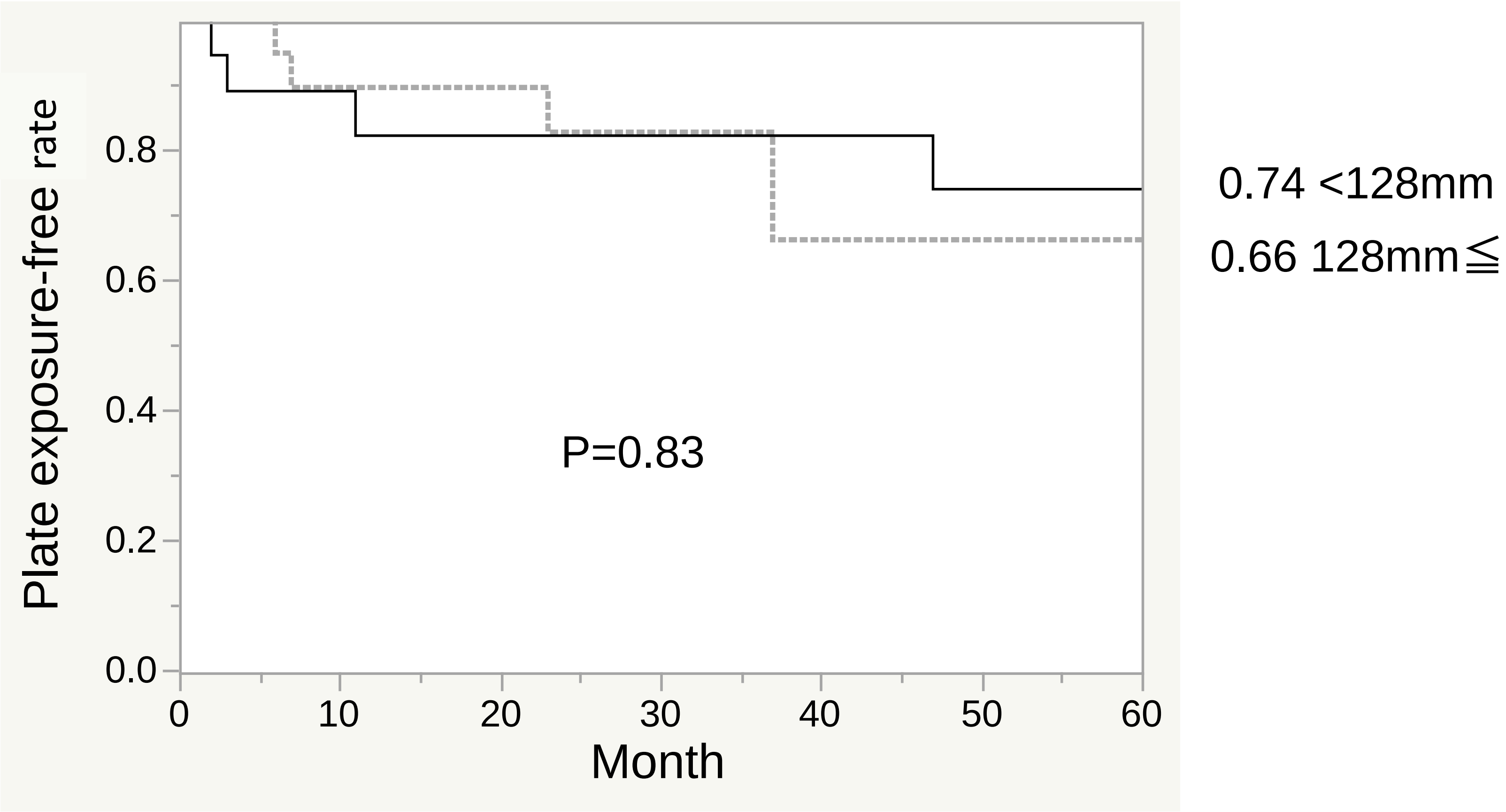
Plate exposure-free rate
0.8
0.6
0.4
0.2
0.0
0
10
20
30
40
50
60
Month
P=0.83
0.74 <128mm
0.66 128mm≦

Figure S3

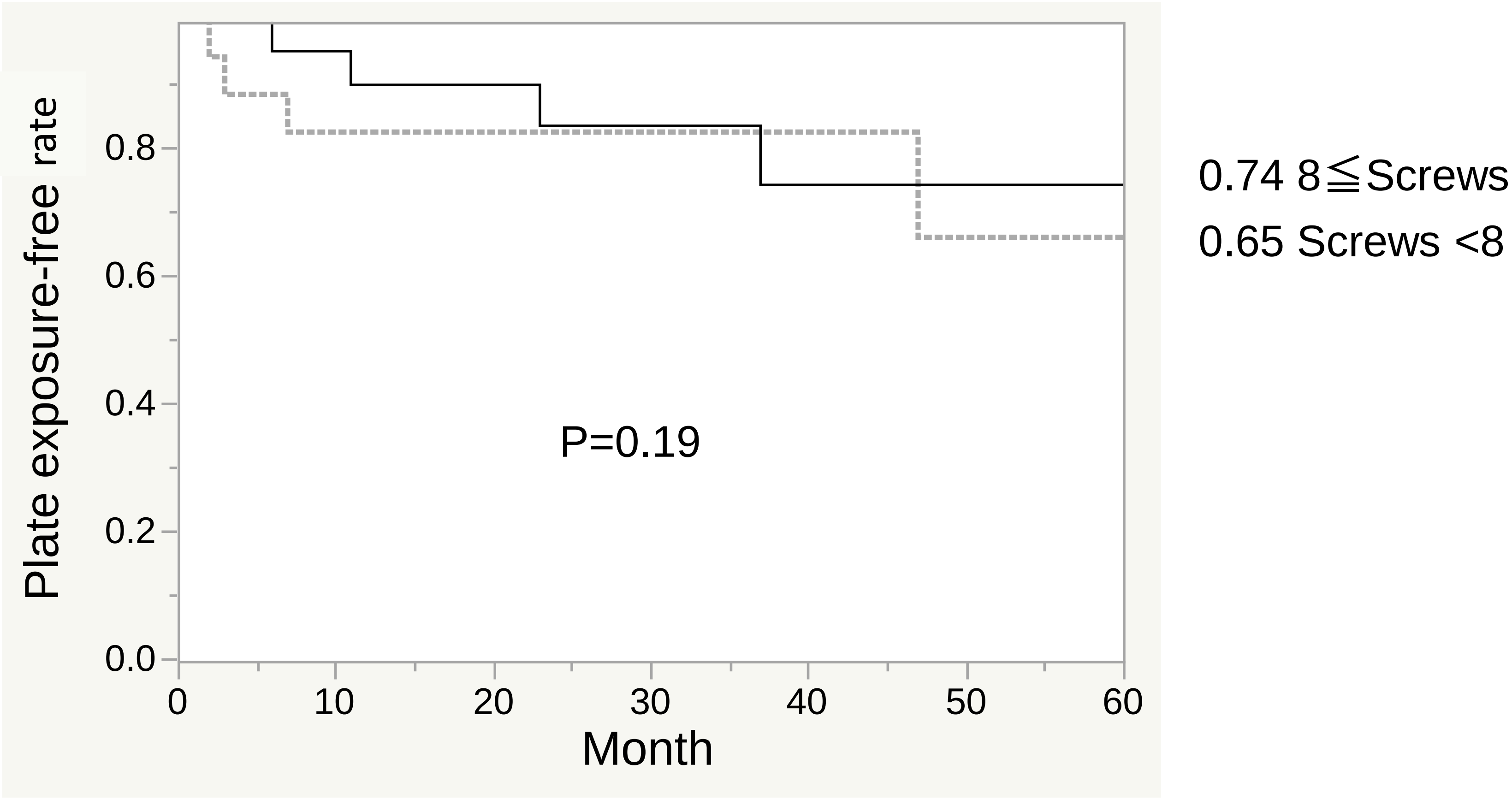
Plate exposure-free rate
0.0
0.2
0.4
0.6
0.8
0
10
20
30
40
50
60
Month
P=0.19
0.74 8≦Screws
0.65 Screws <8

Figure S4

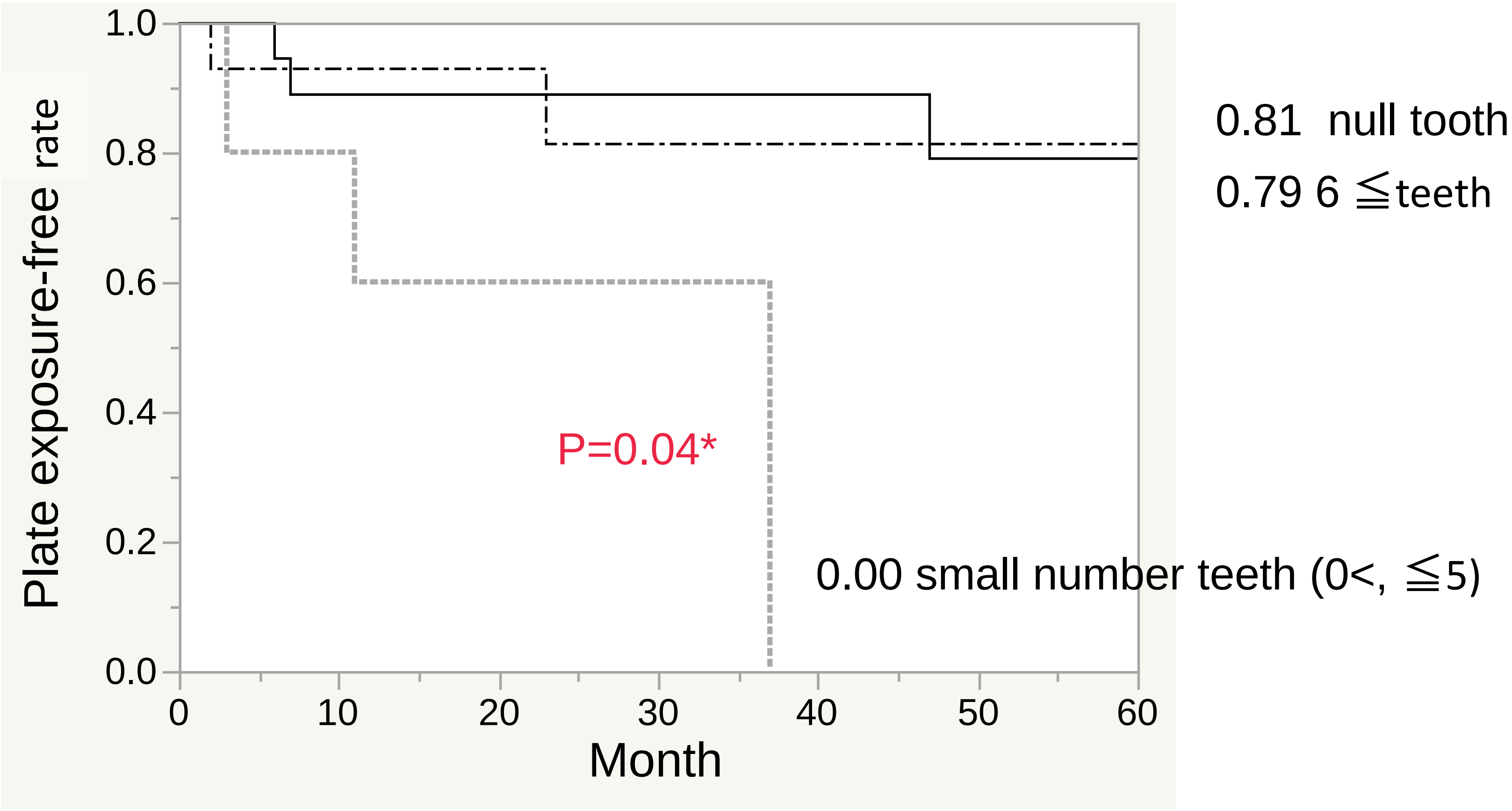

1.0
0.8
0.6
0.4
0.2
0.0
Plate exposure-free rate
0
10
20
30
40
50
60
Month
P=0.04*
0.81 null tooth
0.79 6 ≦teeth
0.00 small number teeth (0<, ≦5)

Figure S5

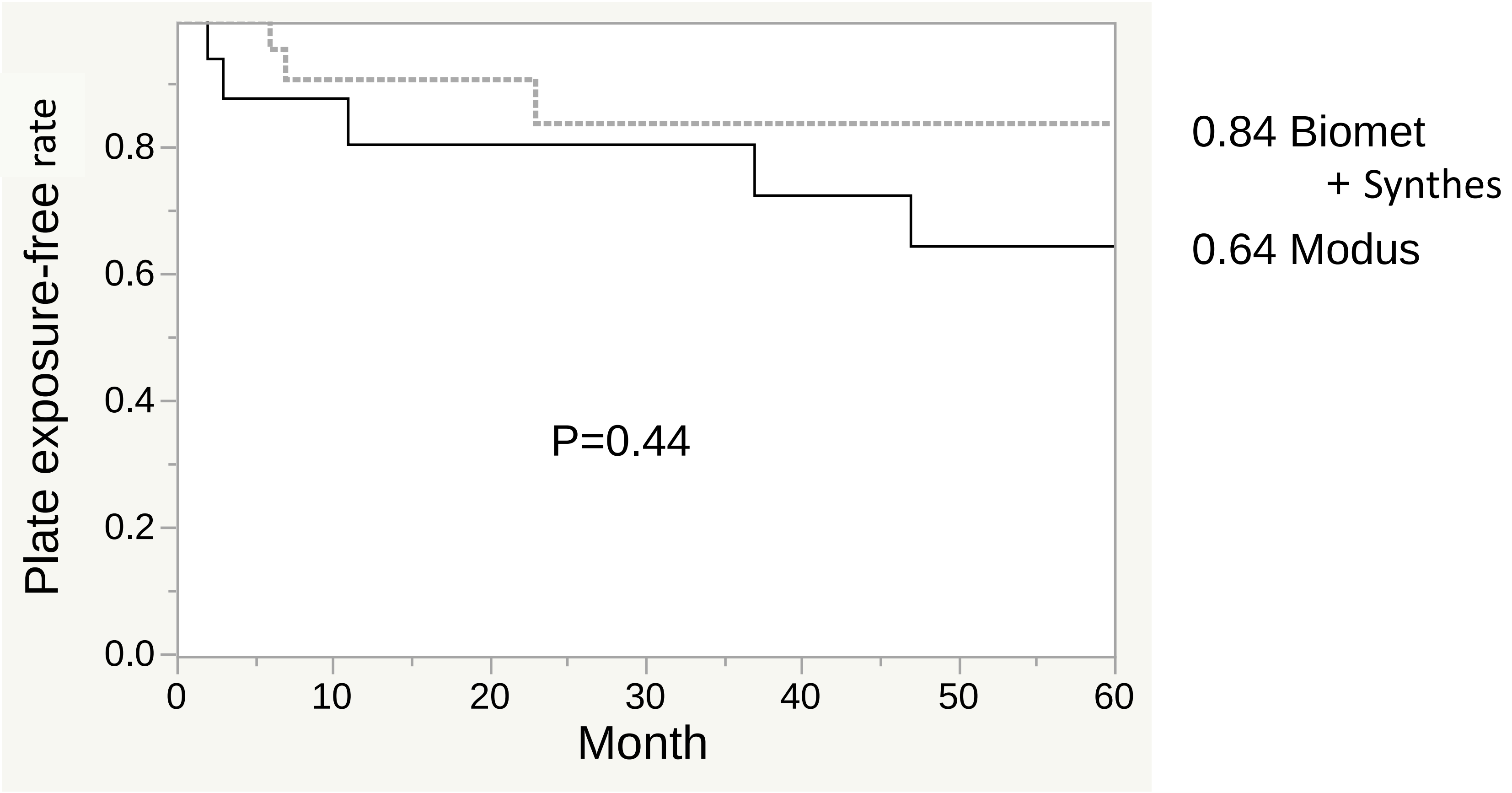

Plate exposure-free rate
0.8
0.6
0.4
0.2
0.0
0
10
20
30
40
50
60
Month
P=0.44
0.84 Biomet
+ Synthes
0.64 Modus

Figure S6

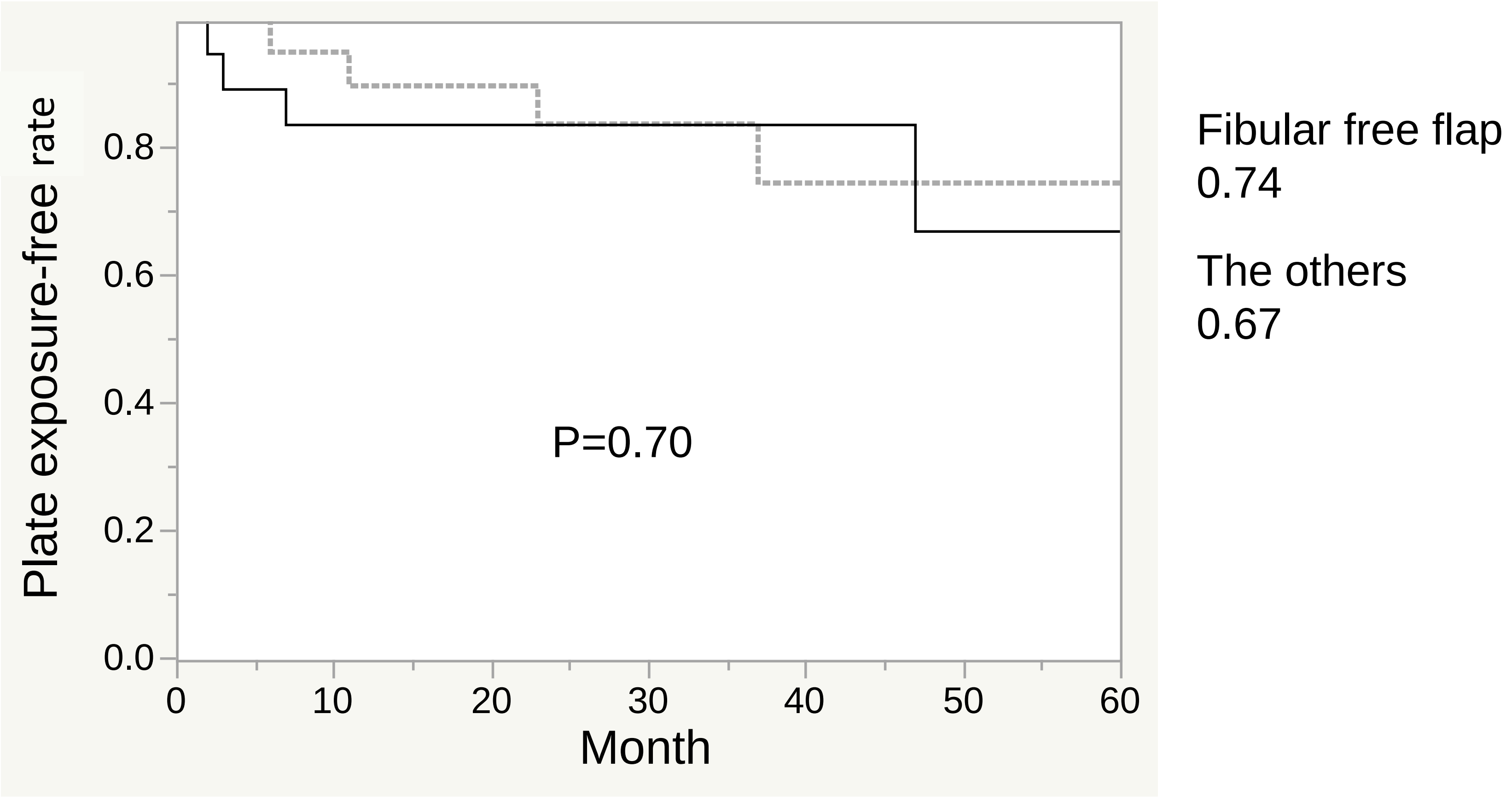
Plate exposure-free rate
0.0
0.2
0.4
0.6
0.8
0
10
20
30
40
50
60
Month
P=0.70
Fibular free flap
0.74
The others
0.67

Figure S7

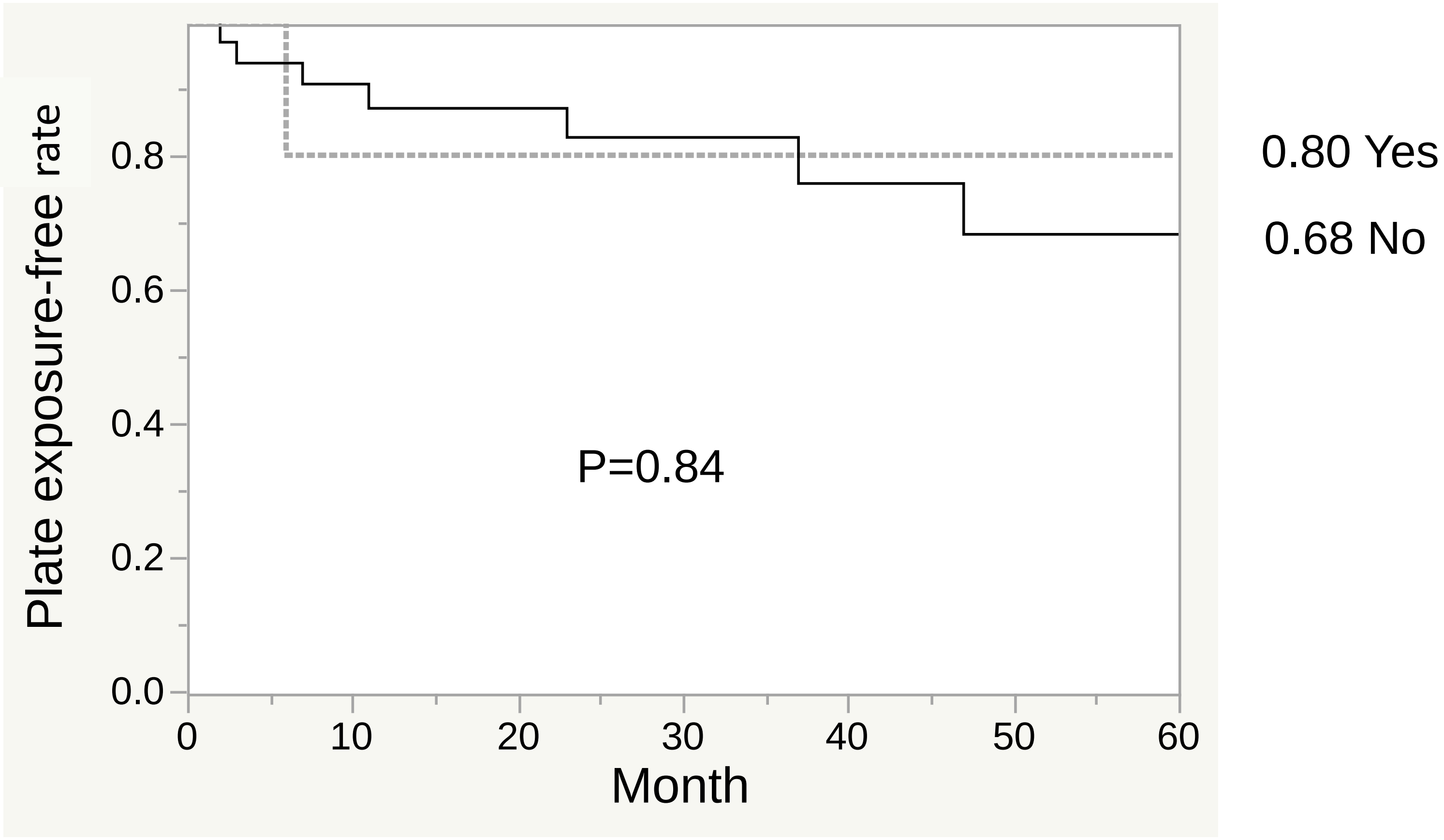

Plate exposure-free rate
0.0
0.2
0.4
0.6
0.8
0
10
20
30
40
50
60
Month
P=0.84
0.80 Yes
0.68 No

Figure S8

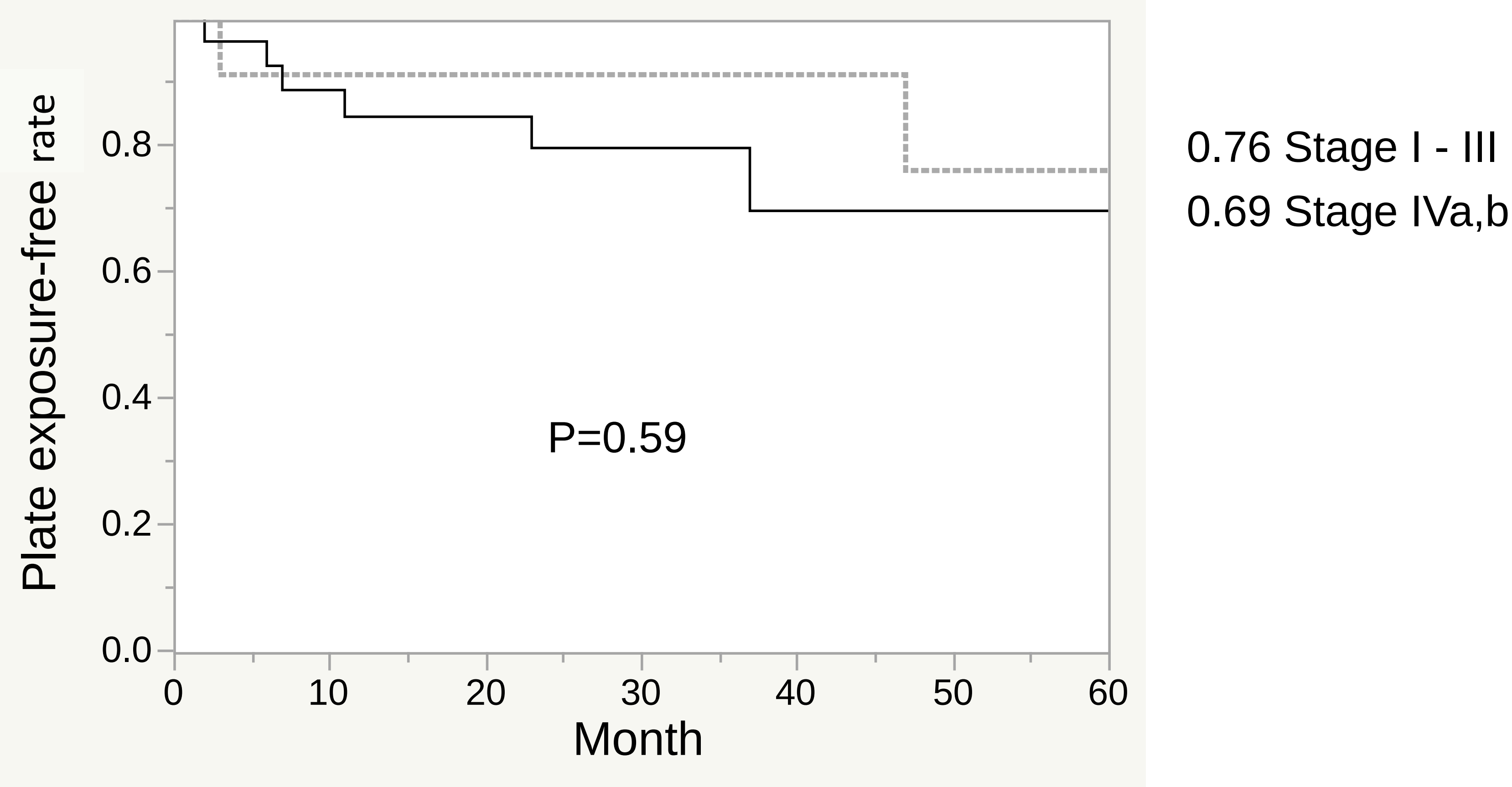
Plate exposure-free rate
0.0
0.2
0.4
0.6
0.8
0
10
20
30
40
50
60
Month
P=0.59
0.76 Stage I - III
0.69 Stage IVa,b

Figure S9

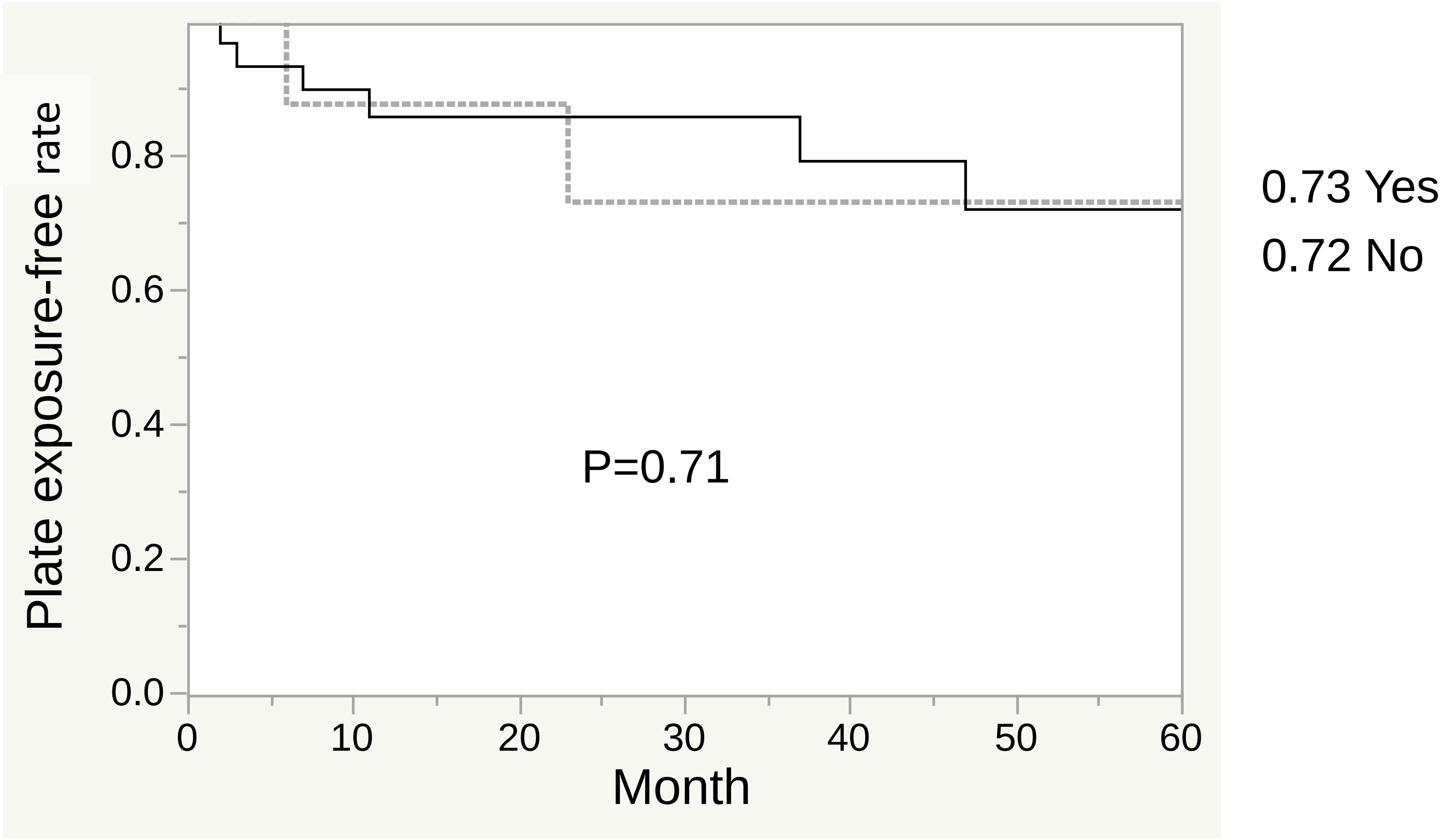
Plate exposure-free rate
0.8
0.6
0.4
0.2
0.0
0
10
20
30
40
50
60
Month
P=0.71
0.73 Yes
0.72 No

Figure S10

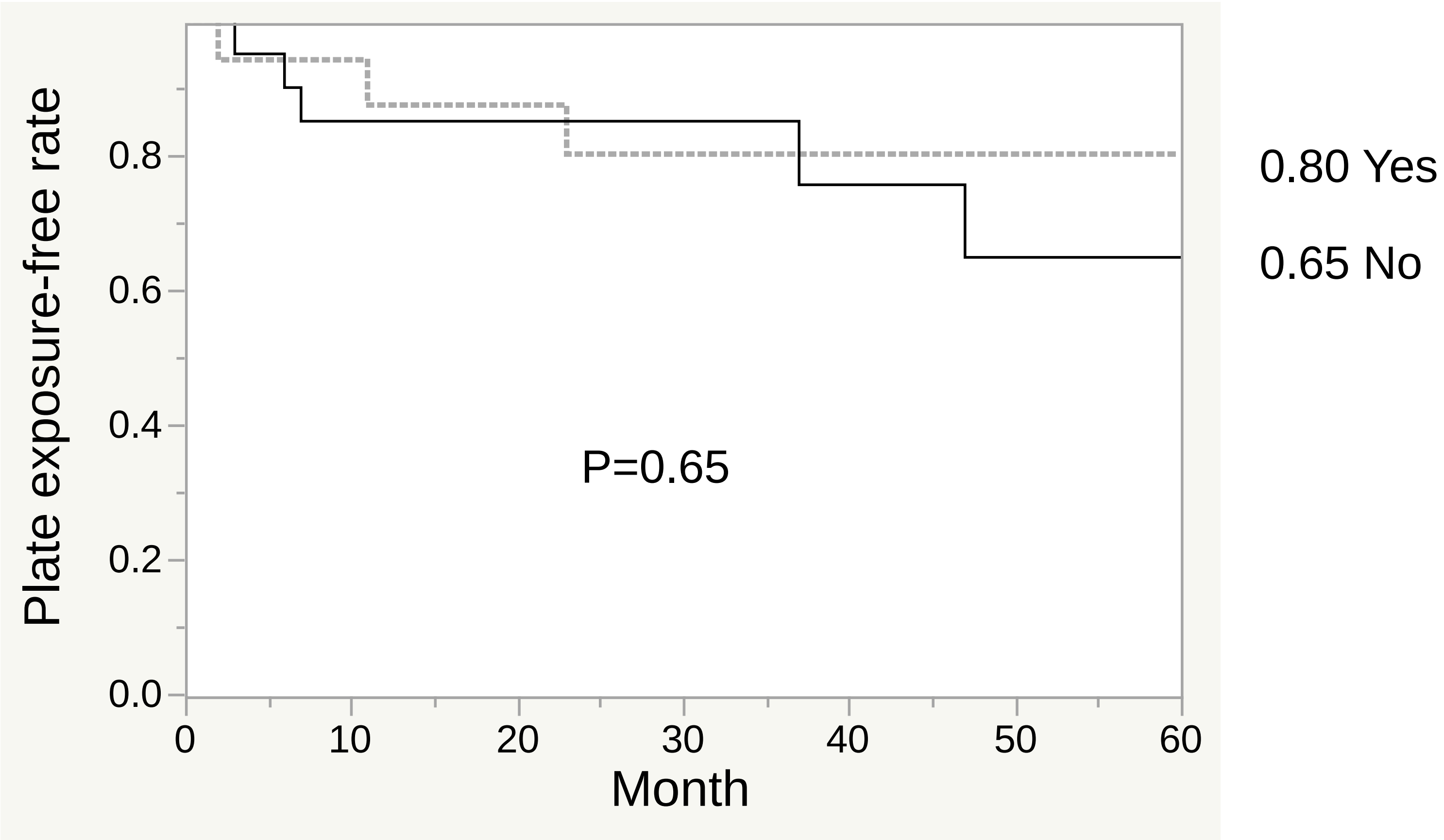

Plate exposure-free rate
0.8
0.6
0.4
0.2
0.0
0
10
20
30
40
50
60
Month
P=0.65
0.80 Yes
0.65 No

Figure S11

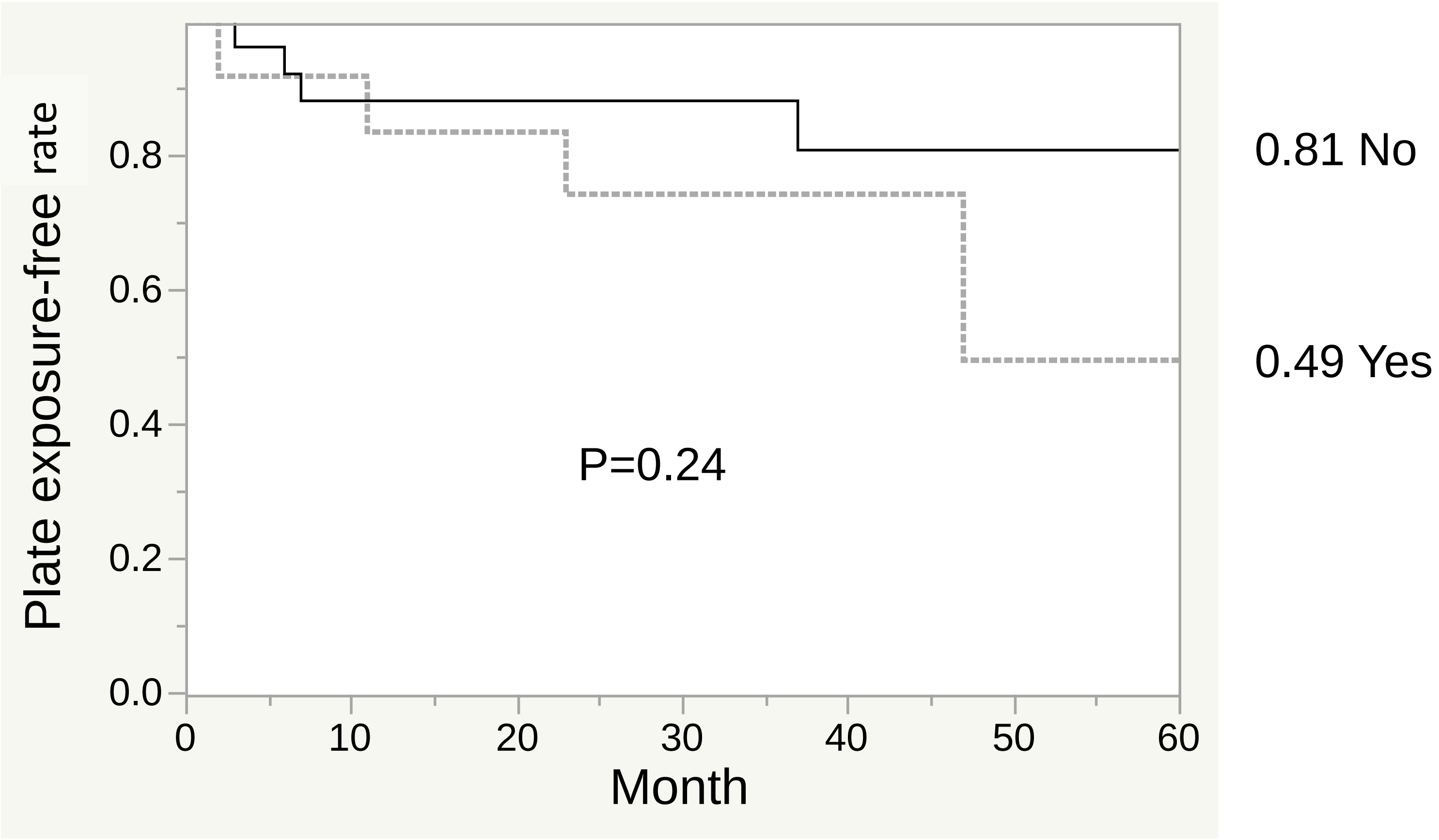

Plate exposure-free rate
0.8
0.6
0.4
0.2
0.0
0
10
20
30
40
50
60
Month
P=0.24
0.81 No
0.49 Yes

Figure S12

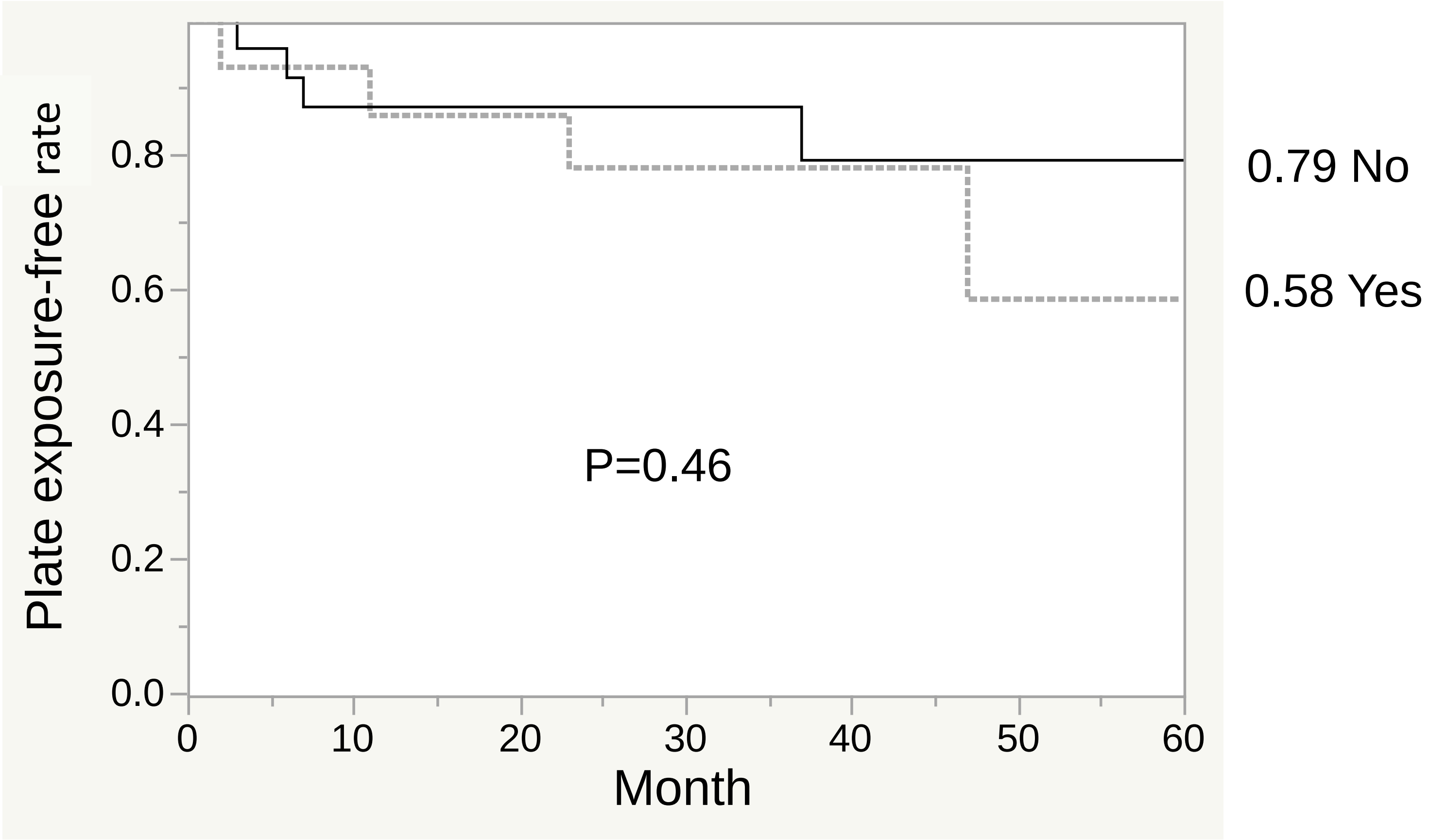
Plate exposure-free rate
0.8
0.6
0.4
0.2
0.0
0
10
20
30
40
50
60
Month
P=0.46
0.79 No
0.58 Yes

Figure S13
